# A Finite Element Analysis Study from 3D CT to Predict Transcatheter Heart Valve Thrombosis

**DOI:** 10.3390/diagnostics10040183

**Published:** 2020-03-26

**Authors:** Francesco Nappi, Laura Mazzocchi, Irina Timofeva, Laurent Macron, Simone Morganti, Sanjeet Singh Avtaar Singh, David Attias, Antonio Congedo, Ferdinando Auricchio

**Affiliations:** 1 Department of Cardiac Surgery, Centre Cardiologique du Nord de Saint-Denis, 93200 Paris, France; 2 Department of Civil Engineering and Architecture, University of Pavia, 27100 Pavia, Italy; laura.mazzocchi02@universitadipavia.it (L.M.); auricchi@unipv.it (F.A.); 3 Department of Imaging, Centre Cardiologique du Nord de Saint-Denis, 93200 Paris, France; dr.irina.timofeeva@gmail.com (I.T.); laurentmacron@gmail.com (L.M.); 4 Department of Electrical, Computer, and Biomedical Engineering University of Pavia, 27100 Pavia, Italy; simone.morganti@unipv.it; 5 Department of Cardiac Surgery, Golden Jubilee National Hospital, Glasgow G81 4DY, UK; sanjeetsinghtoor@gmail.com; 6 Department of Cardiology, Centre Cardiologique du Nord de Saint-Denis, 93200 Paris, France; attias@ccn.fr; 7 Department of Electronic Engineering, AKTIVE Reeds Manufacturing, Computer Science, 80123 Naples, Italy; antonio@aktive.com

**Keywords:** transcatheter implantation, valve replacement, thrombosis, image

## Abstract

Background: Transcatheter aortic valve replacement has proved its safety and effectiveness in intermediate- to high-risk and inoperable patients with severe aortic stenosis. However, despite current guideline recommendations, the use of transcatheter aortic valve replacement (TAVR) to treat severe aortic valve stenosis caused by degenerative leaflet thickening and calcification has not been widely adopted in low-risk patients. This reluctance among both cardiac surgeons and cardiologists could be due to concerns regarding clinical and subclinical valve thrombosis. Stent performance alongside increased aortic root and leaflet stresses in surgical bioprostheses has been correlated with complications such as thrombosis, migration and structural valve degeneration. Materials and Methods: Self-expandable catheter-based aortic valve replacement (Medtronic, Minneapolis, MN, USA), which was received by patients who developed transcatheter heart valve thrombosis, was investigated using high-resolution biomodelling from computed tomography scanning. Calcific blocks were extracted from a 250 CT multi-slice image for precise three-dimensional geometry image reconstruction of the root and leaflets. Results: Distortion of the stent was observed with incomplete cranial and caudal expansion of the device. The incomplete deployment of the stent was evident in the presence of uncrushed refractory bulky calcifications. This resulted in incomplete alignment of the device within the aortic root and potential dislodgment. Conclusion: A Finite Element Analysis (FEA) investigation can anticipate the presence of calcified refractory blocks, the deformation of the prosthetic stent and the development of paravalvular orifice, and it may prevent subclinical and clinical TAVR thrombosis. Here we clearly demonstrate that using exact geometry from high-resolution CT scans in association with FEA allows detection of persistent bulky calcifications that may contribute to thrombus formation after TAVR procedure.

## 1. Introduction

Transcatheter aortic valve replacement (TAVR) is now a common cardiac operation performed worldwide. It is the most effective aortic implantation method for several categories of patients affected by severe aortic valve stenosis [1,2,3,4,5,6,7]. Although transcatheter aortic valve procedures have been performed for more than 17 years [8], no detailed guidelines on the choice of TAVR for younger low-risk patients have been published [9,10], and the choice of strategy remains more an art than a science [11]. Moreover, there is a clear contradiction between the proven benefits of transcatheter heart valve therapy (THVT) and a disguised concern related to thrombosis and early structural valve deterioration [12,13]. 

Subclinical leaflet thickening, reduced leaflet motion of bioprosthetic aortic valves and manifested thrombosis in transcatheter aortic valve replacement are more common than previously appreciated [14,15,16,17,18]. Recently, the GALILEO-4D Clinical Trial [19] showed that the precise causative mechanisms remain unknown despite the use of rivaroxaban-based antithrombotic strategy (10 mg rivaroxaban plus 75 to 100 mg aspirin once daily) or an antiplatelet-based strategy (75 mg clopidogrel plus 75 to 100 mg aspirin once daily). 

Vollema et al. [16] focused attention on the phenomenon of early hypo-attenuated leaflet thickening (HALT) documented either in standard aortic valve replacement or in TAVR [14], which is a cause for concern in both procedural effectiveness and durability of the device. The HALT could serve as a promoter for leaflet thrombosis leading to increased transvalvular gradients, an influencing determinant of early structural valve deterioration and an increased risk for ischemic accidents [17]. The single-center study by Nührenberg et al. [20] focused on the phenomenon of hypo-attenuated leaflet thickening as a potential precursor of thrombosis. The authors evaluated the association between platelet reactivity and HALT following transcatheter aortic valve replacement. All patients, including those with oral anticoagulation treatment, had dual antiplatelet therapy with aspirin and clopidogrel for at least 24 h prior to the procedure. In patients who had pre-existing indications for oral anticoagulation treatment, aspirin was discontinued, but the therapy was continued after TAVR for all the rest. Recipients of the protocol were checked for platelet function, and 4D computed tomography was performed five days after valve implantation to determine the association between baseline platelet reactivity and hypo-attenuated leaflet thickening. The most important finding of this study showed an 18% incidence of hypo-attenuated leaflet thickening with lower complication rates for patients treated with oral anticoagulation. Authors concluded that patients with dual antiplatelet therapy (aspirin and clopidogrel) did not experience a change in the onset of early hypo-attenuated leaflet thickening. 

It is indisputable that these complications may have an effect on everyday clinical practice. In the hope of encouraging wider diffusion of THVT and to provide a guide for clinicians, we have developed a predictive model to evaluate the progression of thrombotic process with the aim to discuss current evidence for the use of this operation. We propose a research investigation using high-resolution biomodelling from computed tomography scanning. Calcific blocks were extracted from a 250 CT multi-slice image for precise three-dimensional geometry image reconstruction of the root and leaflets.

## 2. Material and Methods

### 2.1. Study Design and Oversight

The study was a retrospective cohort analysis conducted on 98 patients who received the commercially available self-expanding TAVR. First-generation CoreValve self-expandable catheter-based aortic valve replacements were implanted in 82 patients. In the remaining patients, 7 received second-generation self-expandable catheter-based replacements and 9 received the Portico device (ethics approval: IRB CCN_TAVR_1-1218, 29 December 2018). 

### 2.2. Patient Selection

The selected patients underwent TAVR from November 2014 to April 2018 at the Centre Cardiologique du Nord. The eligibility criteria for the study was the implantation of self-expanding transcatheter aortic valve replacement in patients who had developed symptomatic thrombosis after implantation of the device at one year. Eligible symptomatic patients were identified and re-hospitalized with New York Heart Association Class (NYHA) class IV symptoms and severe stenosis of device. Diagnosis of thrombosis was confirmed at the CT scan.

### 2.3. CT Imaging and Evaluation

**Figure 1:**
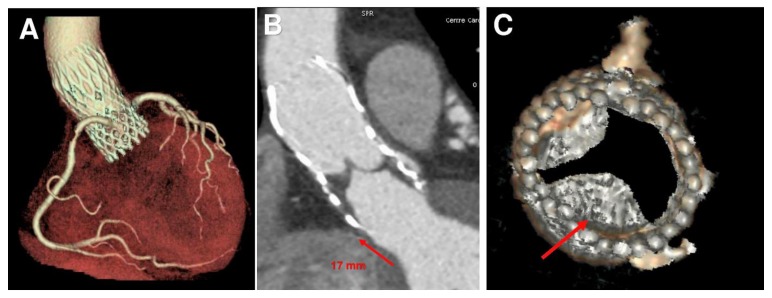
Transcatheter aortic valve replacement (TAVR) position, thrombus formation and biomechanical model of patient-specific aortic root from medical CT images. (**A**) Pre-operative CT scan of CoreValve (26 mm). (**B**) Post-operative CT scan at one year shows a device upward shift. (**C**) Thrombus formation in subvalvular zone (red arrow).

**Figure 2:**
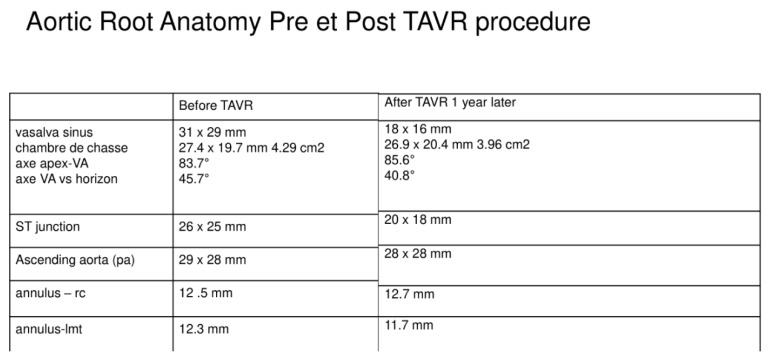
Aortic root anatomy pre et post TAVR procedure. TAVR caused by the TAVR movement. The dimensions are changed for the displacement of device.

**Figure 3:**
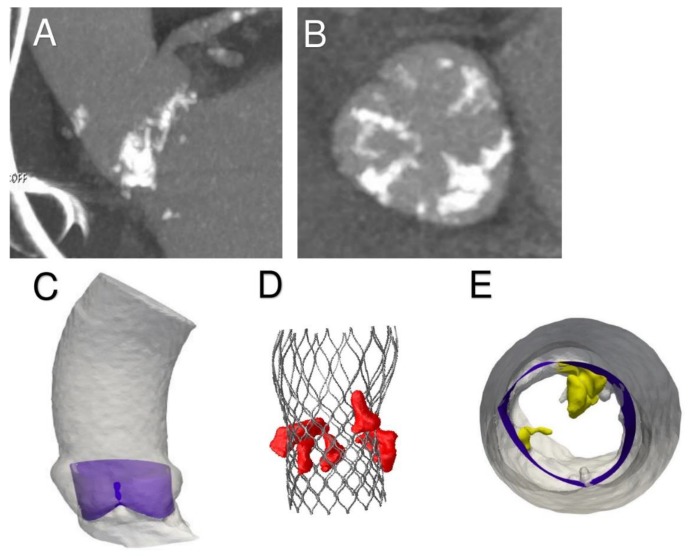
(**A**,**B**) Preoperative CT scan shows massive calcifications of aortic leaflet and root. (**C**) Extraction of aortic root geometry. (**D**,**E**) 3D preoperative model: aortic wall with leaflets and calcific blocks.

**Figure 4:**
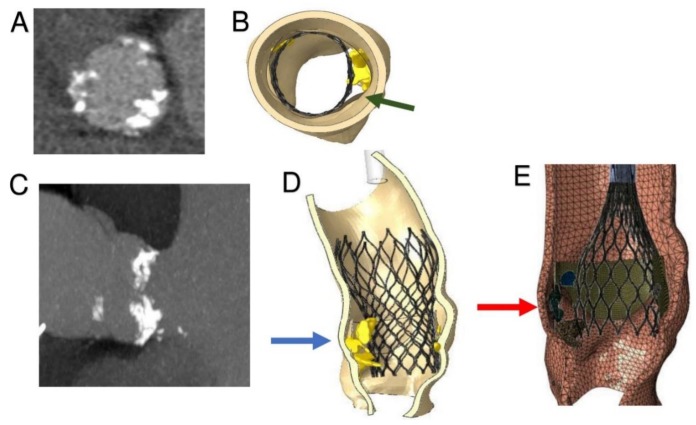
Inclusion of calcific blocks and Finite Element Analysis simulation of TAVR procedure. (**A**) CT image, aortic valve from transverse plane: leaflet calcifications. (**B**) Top view of patient-specific 3D aortic root reconstruction: calcifications attached to the leaflets. Persistent bulky calcifications may determine the development of paravalvular leakage. (**C**) Influence of bulky calcification on device biomechanical behavior. (**D**) Simulation of stent self-expansion after catheter removal: lack of prosthetic anchorage corresponding to uncrushed calcific plaques (blue arrow). (**E**) Great calcification prevents good left ventricular outflow tract (LVTO) inferior placement of CoreValve (26 mm) and upward stent migration (red arrow).

**Figure 5:**
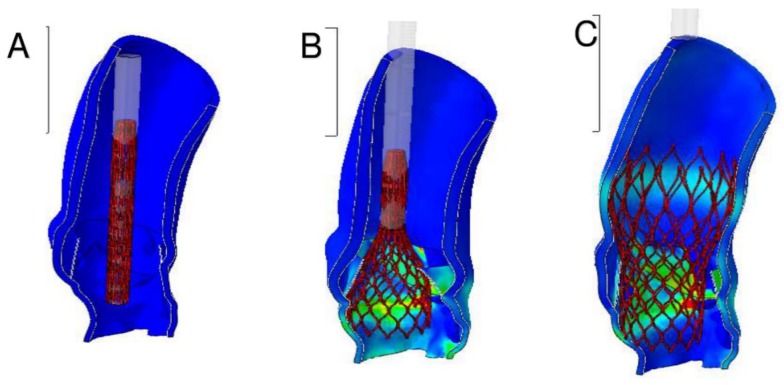
Results of FEA: evaluation of von Mises average stress distribution induced by the device expansion along the native aortic structure during and after implant simulation. (**A**) Stent crimping. (**B**) Catheter removing. (**C**) Stent self-expansion.

**Figure 6:**
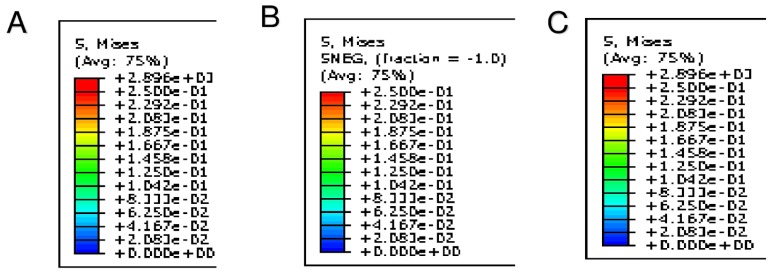
Numerical values expressed in MPa. (**A**) Stent crimping. (**B**) Catheter removing. (**C**) Stent self-expansion.

**Figure 7:**
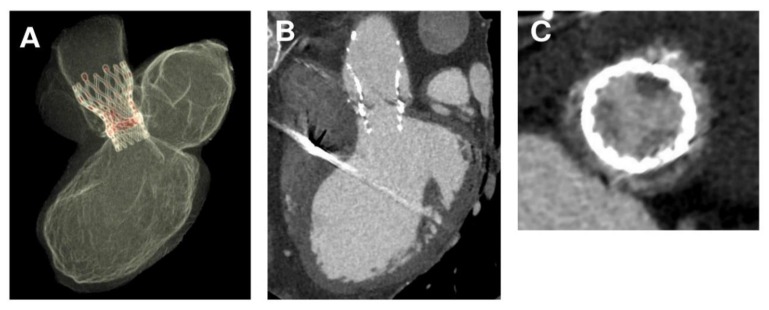
(**A**–**C**) Valve-in-valve thrombosis of CoreValve self-expanding TAVR.

**Figure 8:**
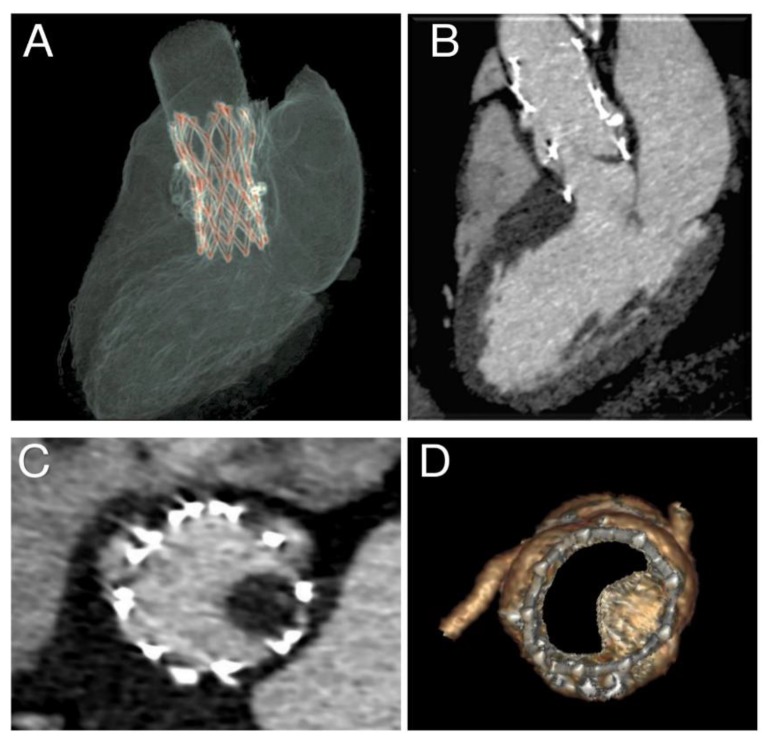
(**A**–**D**) Thrombosis of 29 mm self-expandable Portico device.

All 98 patients had preoperative CT scans. Our institutional TAVR protocol does not mandate systematic CT scans post-TAVR in patients with uncomplicated postoperative courses. Thus, only the 39 patients with unplanned heart failure rehospitalization underwent CT at varying intervals after catheter-based aortic valve replacement. Of a total of six devices affected by symptomatic THVT, three patients were carriers of self-expanding TAVR. Two of those patients received a first-generation CoreValve device. As stated in Figure 1, Figure 2, Figure 3, Figure 4, Figure 5, Figure 6 and Figure 7, the sizes were 26 and 23 mm. The third patient received a Portico self-expandable catheter-based aortic valve replacement. As stated in Figure 8, the size was 29 mm. 

All 3D CT scan procedures were obtained with the 256-row multi-slice GE using volume-rendered CT-acquisition protocol (General Electric Healthcare, Chicago, IL, USA). Qualitative and quantitative assessment of left ventricular outflow tract, aortic root, ascending aorta, and leaflet morphology were performed. Finally, leaflet motion with volume-rendered en-face CT imaging of the native aortic valve and TAVR prosthesis at maximal systolic leaflet opening was quantitatively assessed. 

### 2.4. The Use of Finite Element Analysis Investigation

Presently, the mechanism of THVT cannot be directly predicted, but it can be determined through finite element analyses (FEAs). By means of FEA, we obtained knowledge about complicated real-world systems that would otherwise be impossible to directly determine. First, we applied FEA to medical device designs, and we used this pivotal procedure to calculate stresses and investigate potential failure modes and locations. Second, we performed a predictive biomechanical modelling of aortic root and leaflet through the FEA to obtain specific features directly extracted from CT imagery of the patient’s aortic root preoperatively. Our goal was to determine the risk of THVT in the first generation of TAVR (Medtronic, Minneapolis, MN, USA). In addition, non-cylindrical trans-aortic valve (TAV) shape after implantation in the calcified aortic root, which can cause paravalvular regurgitation, was observed. 

### 2.5. Computed Biomodelling of Thrombosis

The patients with severe calcified aortic stenosis who underwent a TAVR procedure with a self-expanding device were studied with the 256-row CT scan. The adopted computational framework to simulate transcatheter aortic valve replacement was used to investigate the first commercially available 26 mm CoreValve self-expandable catheter-based aortic valve replacement, which was the most widely used self-expandable device with 82 procedures. 

The FEA evaluation can be categorized into four main phases: Phase 1 was the processing of medical images. Phase 2 was the establishment of suitable models for analysis. Phase 3 was the simulation of the entire clinical procedure after analysis of the acquired data. Phase 4 was the post-processing of the simulation results and comparison with follow-up data.

The commercial finite element solver Abaqus 6.14 by Dassault Systèmes (Simulia, Providence, RI, USA) was used to create an aortic valve Finite Element Model (FEM) and perform all simulations—stent crimping and prosthesis implantation in the native root. Preoperative CT images were initially used to create a patient-specific geometrical model of the aortic valve complex consisting of aortic root wall, native leaflets, and calcific plaques. Details on the FEA methodology are reported in the Supplementary Materials.

## 3. Results 

Thrombotic formation with 26 mm CoreValve transcatheter valve therapy (online Video 1) was detected one year after implantation. 

### 3.1. Phase 1—Processing Medical Images

Echocardiography showed high transvalvular gradients (peak 34 mm Hg; mean 23 mm Hg; dimensionless valve index 0.23), mild central leakage, and elevated left ventricular dimensions with normal left ventricular function. Thrombotic formation was detected at transthoracic echocardiography (TTE) and subsequent transoesophageal echocardiography (TEE) (online Video 2). 

CT scan showed thrombosis of the inner surface of the CoreValve where a thrombus measuring 20 × 15 mm was located in the subvalvular zone. The thrombus was at the level of the posterior and anterior right leaflet and presented with extensions into the supravalvular zone, rendering the posterior leaflet quasi-totally blocked in closed position with a measured planimetry of 1.5 cm². The coronary ostia were free of lesions, and there was no evidence of embolism (online Video 3). The thrombus extended to the outer surface of the CoreValve involving the sinus of Valsalva. The device had migrated 17 mm cranially (Figure 1A–C and Figure 2). The patient was promptly commenced on anticoagulant therapy. Seven days after treatment, CT imaging revealed a residual partial extension of subvalvular thrombus with a marked regression of the circumferential supravalvular mass partially limited to the right and left aortic sinus. The thrombus was predominantly organized on the posterior leaflet as a nodular formation of 4–4.5 mm (online Video 4). Both TTE and TEE revealed a significantly reduced transvalvular gradient of 10–11 mm Hg with no evidence of central leakage (online Video 5). The patient was discharged home on anticoagulant therapy. Upon follow-up four weeks later, the images revealed further regression of thrombus (online Videos 4 and 5).

### 3.2. Phase 2—Simulation of the Entire Clinical Procedure after Analysis of the Acquired Data

Pre-operative CT images (Figure 3A,B) were used to create an accurate geometrical model of the aortic valve complex of the patient consisting of aortic root wall, native leaflets, and calcific plaques (Figure 3C–E; Figure 4B,D,E). Based on the reconstructed geometrical model, Finite Element Analysis (FEA) was performed using the commercial FE solver Abaqus 6.14 by Dassault Systèmes (Simulia, Providence, RI, USA), following the detailed procedure described by Morganti et al. [21] (Supplementary Methods). CT images taken after stenting have been used to validate our simulation outcomes by direct comparison with post-operative data. During the FEA simulation of TAVR procedure, von Mises average stress distribution was computed to measure the stress induced by the device expansion onto the inner wall of the aortic root (Figure 4B,D,E; Figure 5A–C; Figure 6A–C). 

### 3.3. Phase 3—Post-Processing of the Simulation Results and Comparison with Follow-Up Data

As shown in the image study results, the location of stress peaks corresponded to the sites of higher global anchoring forces. Therefore, as expected, greater values of stress were associated with the contact areas between the stent frame and the patient-specific aortic structure (0.1667–2.715 MPa). Conversely, lower values were revealed in correspondence with the position of refractory bulky calcifications, noted after deployment of the self-expanded valve, which could not cover the entire circumference of the annulus, leaving a large paravalvular orifice (Figure 4B, green arrow). The device was not properly aligned with the aortic root, thereby lacking complete basal attachment and showed stent deformation (Figure 4D, blue arrow). Evidence that TAVR biomechanical behavior was influenced by calcific blocks attached to the leaflets is shown in Figure 3A,B and Figure 4A,C. Compared to the shape before the crimping procedure (Figure 2), a qualitative evaluation of real stent deformation in the investigated patient’s root clearly highlights an incomplete and asymmetric expansion of the device (Figure 4B,D).

## 4. Discussion

Just a few years ago, the heart surgery community experienced a reversal of the trend of standard operation for aortic valve replacement, and there was a drastic reduction in the number of mechanical prostheses implanted compared to stented xenografts [22,23] because thrombosis in surgical bioprosthesis occurs more rarely (1%–2% of recipients) [24], which is significantly less compared to the modern-day TAVR [17,23]. Today, in the transcatheter heart valve therapy scenario, one of the exciting arguments for optimizing the TAVR procedure is improved antiplatelet therapy, although clinical investigators are enticed by novel anticoagulant drugs [25]. 

Transcatheter heart valve thrombosis is an ongoing thorn in the effectiveness of long-term outcomes for the procedure. The importance of post-TAVR thrombotic complication as initially described by Hanson showed a 7% THVT rate with 18% of patients with clinically obstructive TAVR thrombosis despite the administration of dual antiplatelet medication [15]. However, concerns emerged prematurely in the results of Placement of AoRTic TraNscathetER Valve Trial Partner II and III randomized clinical trials, and widespread empirical use of dual antiplatelet therapy was supported by evidence of 75% of recipients of catheter-based aortic valve replacement developing embolic detritus [3,5]. In the last two years, investigators have tried to provide an answer to the problem by primarily optimizing antithrombotic therapy with aspirin alone or aspirin plus clopidogrel administration, thus ignoring biomechanical studies on the aortic root post-TAVR procedure. Nührenberg’s work revealed that antiplatelet therapy was ineffective in the prevention of thrombosis, highlighting that an improvement in the understanding of HALT phenomenon after transcatheter heart valve replacement may circumvent the inherent risks of continuous anticoagulant therapy in patients who have received a bioprosthesis. Recently, a GALILEO RCT (global study comparing a rivaroxaban-based antithrombotic strategy to an antiplatelet-based strategy after transcatheter aortic valve replacement to optimize clinical outcomes) showed that a rivaroxaban-based antithrombotic strategy was more effective than an antiplatelet-based strategy in preventing subclinical leaflet motion abnormalities. These results are promising to establish an effective optimal medical treatment and avoid THVT; however, in the main trial, the rivaroxaban-based strategy was associated with a higher risk of death or thromboembolic complications and a higher risk of bleeding than the antiplatelet-based strategy [19].

### Dynamic of Aortic Root and Persistent Bulky Calcification: Still Relevant?

The performance of the percutaneous aortic valve procedure is affected by the degree of native valve calcifications, stent deformation, the size of the patient’s annulus, and a physiological dynamic of blood [26]. 

First, the presence of calcified blocks in the annulus raises questions on the indication of the use of stented prostheses that may lead to geometric transformations of the aortic annulus after the deployment of the device [21,27]. Balloon and self-expandable catheter-based aortic valves may be ineffective on solid and bulky native aortic valve calcifications. In our own study, the Sapien XT showed high values of the maximum principal stress in the aortic regions closed to solid calcific blocks resulting in the deformation of the stent which assumes an elliptical shape [27]. The consequence of the geometric modification in the more accentuated form can lead to leaflet mal-coaptation due to paravalvular leakage, while in the lesser accentuated ones it can lead to hypo-attenuated leaflet thickening. We hypothesize that the second, less evident elliptical deformation potentially predisposes patients to subclinical thrombosis due to the presence of residual bulky native calcification favoring hypomobility [21,27]. In self-expanding devices, we have demonstrated the crucial role of positioning in determining valve anchorage. Non-uniform expansion related to extensive calcifications is responsible for prosthetic device deformation that leads to an eccentricity > 10%, resulting in incomplete expansion of the metallic frame at almost all levels [21].

Second, the current progress of technology and design associated with advanced clinical studies probably continues to collide with the concept that transcatheter heart valve replacement was initially conceived for use in the pulmonary artery [28], which has a higher degree of extensibility and distortion with respect to the aortic root [29,30,31,32,33,34,35]. This delicate peculiarity does not apply to the Valsalva sinus of the aortic root, where the predisposition to receive material such as stents is different in both nitinol constituents of the self-expanding valves [21] or cobalt-chromium that integrates balloon-expandable devices [27]. Thus, thrombus formation may be due to the frame of the prosthesis or stasis in the sinus of Valsalva. Currently, the pathophysiological events responsible for transcatheter heart valve thrombosis remain elusive. The process is likely mediated by the formation of platelets or thrombin-related clots [36,37,38].

Third, focusing on antithrombotic management following TAVR may not resolve thrombotic complications. The changes in the dynamic anatomy of the root should also be studied alongside the pharmacodynamics of antiplatelet drugs. Concerns regarding variable pharmacodynamic effects of clopidogrel-based dual antiplatelet therapy remain and may lead to the use of more effective antiplatelet agents such as prasugrel or ticagrelor, which could provide better antithrombotic effects by avoiding the development of HALT. The findings from ongoing trials such as ATLANTIS (Anti-Thrombotic Strategy After Trans-Aortic Valve Implantation for Aortic Stenosis), AUREA (Dual Antiplatelet Therapy Versus Oral Anticoagulation for a Short Time to Prevent Cerebral Embolism After TAVI), ENVISAGE-TAVI AF (Edoxaban Compared to Standard Care After Heart Valve Replacement Using a Catheter in Patients With Atrial Fibrillation), POPular-TAVI (Antiplatelet Therapy for Patients Undergoing Transcatheter Aortic Valve Implantation), and CLOE (Clopidogrel to Lower Adverse Ischemic Events After Transcatheter Aortic Valve) should be integrated with predictive studies on mechanical modelling using computed finite element analysis (FEA) research and 4D CT scan reconstruction. This way, we will not only limit ourselves to antithrombotic treatment optimization but investigate other variables involved in the thrombotic process.

Finally, the application of Finite Element Analysis research to biological structures may be of value [21,27,39,40]. FEA investigation uses the geometric algorithmic prediction to calculate the stress and strain coefficients of the complex. This function is expressed within a small geometric area whereby its behavior can be mathematically anticipated [41]. Although FEA research has been used for more than two decades to study heart valves [10,42,43,44,45,46], its widespread usage in cardiology and cardiac surgery is limited because of the presumed unreliable analysis. Clinicians are hesitant due to the lack of related clinical studies, thereby curtailing its potential effectiveness. Finite Element Analysis biomodelling allows the development of predictive models suitable for investigating the complications related to the TAVR procedure. Therefore, short- and long-term follow-up may be anticipated by means of computed biomodelling applied to TVT. The use of FEA can be clinically validated through a comparative analysis with computed reconstruction of CT scans, magnetic resonance, or 3D TEE reconstruction of the aortic valve [21,27,36], a finding that offers a mathematical physical process to explain the observed advantage in clinical outcomes [10,41,42,43,44,45,46].

## 5. Conclusions

The shift toward routine use of oral anticoagulation administration may be premature considering the availability of bioprostheses, especially since these drugs are widely used by patients over 60 and in some younger patients as well. One of the main reasons for the reaffirmation of stented bioprosthesis in conventional aortic valve surgery is to prevent patients from receiving lifelong oral anticoagulation treatment, as efficacy and safety of the novel oral anticoagulation medications remain unknown, and some studies have even suggested a detrimental association [47]. The use of a predictive model based on the association between CT scan and biomodelling can be useful in selecting patients who would not benefit from TAVR.

## Supplementary Materials

The following are available online at https://www.mdpi.com/2075-4418/10/4/183/s1, Methods for Predictive Biomechanical Modelling; Additional Reference; Online Video 1: TAVR was performed on an 84-year-old woman of prohibitive surgical risk; The video shows the success of the implanted CoreValve (26 mm) with restoration of adequate surface of aortic area. Online Video 2: 1 year later, TTE showed increased mean systolic gradient at 23 mm Hg, central leakage and elevated left ventricular dimensions with normal left ventricular function. TTE and TOE detected thrombus formation; Online Video 3: Postoperative CT scan at 1 year shows obstruction of TAVR. See the thrombus at the level of the posterior and anterior right leaflet with partial extensions in supravalvular zone; the posterior leaflet quasi-totally obstructed in closed position with a planimetry of 1.5 cm²; the thrombus is extended to the outer surface of the CoreValve involving the sinus of Valsalva. The coronary ostia is free of lesions, and there is no suggestion of embolism; Online Video 4: CT scan after anticoagulant medication shows normal cusps and normal gradient. Marked regression of the circumferential supravalvular mass partially limited to the right and left sinus of Valsalva. The thrombus predominantly organized on posterior leaflet as a nodular formation (4–4.5 mm); Online Video 5: One- and four-week TTE and TOE after administration of anticoagulant therapy showed regression of thrombus.
