# Supplementary material for: A Finite Element Analysis Study from 3D CT to Predict Transcatheter Heart Valve Thrombosis
Source: Diagnostics (Basel). 2020 Mar 26;10(4):183. doi: 10.3390/diagnostics10040183 (PMC7235717; doi:10.3390/diagnostics10040183)
Supplement: Supplementary file 1 [file diagnostics-10-00183-s001.zip › Supplementary Materials/diagnostics-742734 supplementary method and reference.pdf]

Supplementary Materials

Methods for Predictive Biomechanical Modelling

The adopted computational framework to simulate transcatheter aortic valve implantation (TAVI) can be divided into four main phases: Step 1, processing of medical images (Figure 1C,D); Step 2, construction of FE models suitable for analysis (Figure 1E,F); Step 3, simulation of device implantation (Figure 2C,D); Step 4, post-processing of the FE calculations (performed to extract quantitative measures of prosthetic stent deformation) and comparison with follow-up data.

The aortic root model is generated by considering a constant thickness of its wall at 2.5 mm, while native leaflets are reconstructed under the assumption of a uniform thickness of 0.5 mm, for simplicity.

To characterize leaflet tissues, we used a linear isotropic elastic model, and simplified St.Venant–Kirchhoff material properties were considered, with Young's modulus E of 2 MPa and a Poisson's ratio υ of 0.45. The hyperelastic material model, described by a six-order reduced polynomial constitutive model proposed by Martin et al. (5), represents the nearly incompressible nature of the cardiac root tissue. Density ϱ was assumed equal to 1.1×10^{-9} ton/mm^3 for both aortic wall and valvular leaflets (6), while we assigned linear elastic properties to the calcified tissues adopting the following parameters: E = 10 MPa, $\upsilon = 0.35$ and $\varrho = 2 \times 10^{-9}$ ton/mm^3 (7). Frictionless contact is considered between the aortic root and the leaflets, while self-contact is applied to the leaflets. The interaction between calcific blocks and leaflets is defined by means of a kinematic coupling constraint technique, and a frictionless general contact is used to handle the interactions between calcifications and the aortic root inner surface. Material properties of the nitinol alloy are used to model self-expandable CoreValve, according to constitutive laws reported in Auricchio et al. (8). After the device crimping within its delivery system, the simplified catheter defined through a cylindrical rigid surface is gradually removed with a sliding upwards movement to let the stent open and exploit its superelastic behavior.

Additional Reference

1. Martin C., Pham T., Sun W. Significant differences in the material properties between aged human and porcine aortic tissues. Eur J Cardio-Thorac Surg 2011, 40(1):28–34

2. Morganti S., Conti M., Aiello M., Valentini A., Mazzola A., Reali A., Auricchio F. Simulation of transcatheter aortic valve implantation through patient-specific finite element analysis: two clinical cases. J Biomech 2014, 47: 2547–2555

3. Xiong F.L., Goetz W.A., ChongC.K., ChuaY.L., PfeiferS., Wintermantel E., Yeo J.H. Finite element investigation of stentless pericardial aortic valves: relevance of leaflet geometry. Ann Biomed Eng 2010, 38: 1908–1918.

4. Auricchio F., Taylor R.L. Comput Methods Appl Mech Eng 1997, 143(1-2): 175-19
